# Study on the Correlation between Interleukin-27 and CXCL10 in Pulmonary Tuberculosis

**DOI:** 10.1155/2022/2932837

**Published:** 2022-06-22

**Authors:** Jiahui Fan, Yefeng Yang, Liang Wang, Xiaoqian Shang, Li Zhang, Hu Sun, Yujie Ma, Ying Li, Jing Wang, Xiumin Ma

**Affiliations:** ^1^State Key Laboratory of Pathogenesis, Prevention and Treatment of High Incidence Diseases in Central Asia, Clinical Laboratory Center, Tumor Hospital Affiliated to Xinjiang Medical University, Urumqi 830000, China; ^2^Department of Respiratory Medicine, Second Affiliated Hospital of Hainan Medical University, Haikou, Hainan 570100, China; ^3^State Key Laboratory of Pathogenesis, Prevention and Treatment of High Incidence Diseases in Central Asia, Department of Respiratory Medicine, First Affiliated Hospital of Xinjiang Medical University, Urumqi, Xinjiang 830000, China

## Abstract

**Objective:**

To investigate the correlation between interleukin-27 and CXCL10 and other cytokines in pulmonary tuberculosis and to further explore the related miRNAs through bioinformatics.

**Methods:**

Collect the lesion tissue and peripheral blood of pulmonary tuberculosis patients and the peripheral blood of healthy controls. Immunohistochemical staining and qRT-PCR were used to observe the expression of interleukin-27, CXCL9, CXCL10, and CXCL11. Then, predict the key miRNA, qRT-PCR was used to verify the expression of miRNA in the peripheral blood and evaluated the correlation between them.

**Results:**

Both immunohistochemical staining and qRT-PCR indicated that the expressions of IL-27, CXCL9, CXCL10, and CXCL11 were significantly increased in tuberculosis patients, and IL-27 was significantly correlated with CXCL10 (*r* = 0.68). Key molecules such as has-let-7b-5p, has-miR-30a-3p, and has-miR-320b were screened out. Among them, has-let-7b-5p was significantly downregulated, and has-miR-30a-3p was significantly upregulated; they were related to interleukin-27 and CXCL10.

**Conclusion:**

Our data shows that interleukin-27 and CXCL10 are significantly related in pulmonary tuberculosis, and has-let-7b-5p and has-miR-30a-3p are also related to interleukin-27 and CXCL10. It laid the foundation for subsequently exploiting the potential biomarkers in tuberculosis disease.

## 1. Introduction

Tuberculosis (TB) is a disease caused by Mycobacterium tuberculosis (MTB) infection through the respiratory tract. According to the Global Tuberculosis Report released in October 2020, the global incidence of tuberculosis is about 132 per 100,000, and China ranks second among 30 countries with a high burden of tuberculosis with 866,000 cases [1]. Since 2014, although the infection rate of Mycobacterium tuberculosis has been declining year by year, in the prevention and treatment of tuberculosis, some progress has been made [2]; the outbreak of new Coronavirus disease (COVID-19) at the end of 2019, temporary disruptions caused by social closures can also lead to increased TB morbidity and mortality [3, 4]. The misfortune, and even the social and economic burden, that TB brings to patients and their families cannot be ignored [5]. Therefore, it is necessary to explore the pathogenesis of tuberculosis and discover new biomarkers.

For the occurrence and development of tuberculosis, immune inflammation caused by MTB is considered to be an important process, and this dynamic balance of immunization-inflammation has an important impact on the end of the disease. A large number of studies have focused on signaling pathways regulate immune inflammatory responses during MTB infection [6]. Studies have shown that the interaction of MTB and Toll-like receptors activates innate immunity and inflammation, which in turn activates transcription factors to generate proinflammatory factors such as interleukins [7, 8]. They attract or chemoattract inflammatory cells to the lesions where MTB is located and activates cells to secrete IL-8, MCP-1, and CXCL10 and other inflammatory mediators, so that more and more immune cells migrate to the site of infection, form a granulomatous structure, and block the further spread of pathogens in the tissues [7, 8].

Interleukin-27 (IL-27) belongs to the IL-12 family of cytokines. It is composed of p28 and EBI3. When microorganisms infect the body, antigen-presenting cells (APCs) produce IL-27, which acts as an anti-inflammatory [9, 10]. Chemotactic cytokines are proteins which can make immune cells migrate to the lesion sites. CXCL9, CXCL10, and CXCL11 can bind to C-X-C motif chemokine receptor 3 (CXCR3) [11], exert chemotactic effect, and make lymphocyte gather to participate in the immune response of the lesion site.

In previous studies, the high expression of IL-27, CXCL9, and CXCL10 in the peripheral serum, pleural effusion, and bronchial lavage fluid of tuberculosis patients used in the diagnosis and differential tuberculosis has a good performance [12–14]. They are important existences that cannot be ignored. However, just as an independent index to evaluate diagnostic performance is not enough, and we note that studies have shown that IL-27 can activate monocyte lymphocytes and other immune cells to generate CXCL10 and other chemokine, thus promoting the formation of granuloma in tuberculosis patients, to eliminate pathogenic bacteria [15–17]. Therefore, we tried to explore the relationship between IL-27 with CXCL10 and other chemokines, to provide a research basis for further exploration of the interaction between IL-27 with CXCL10.

In this study, we used immunohistochemistry to detect the expression levels of IL-27, CXCL9, CXCL10, and CXCL11 in patients with tuberculosis and observe their expression in the lesion. Then, through bioinformatics methods, further explore the important molecules that are closely related to IL-27, CXCL9, CXCL10, and CXCL11 in the course of tuberculosis, and then, predict miRNA, peripheral blood of tuberculosis patients was collected; the expression of cytokines and miRNA was verified by qPCR. We hoped that potential microRNAs and circular RNAs that play an important role in tuberculosis can be found, so as to lay a certain foundation for revealing the pathogenesis of tuberculosis at the molecular level and dig out the potential biomarkers of tuberculosis.

## 2. Materials and Methods

### 2.1. Participants

43 active pulmonary tuberculosis patients were collected; among them, 23 were male and 21 were female, with an average age of 49.51 ± 11.90 years old. They were diagnosed at the First Affiliated Hospital of Xinjiang Medical University from December 2018 to December 2020, and 5 ml EDTA anticoagulated whole blood was drawn during the active period. At the same time, a total of 37 cases of healthy control group were collected, including 21 males and 16 females, with an average age of 40.27 ± 13.61 years old, collected 5 ml EDTA anticoagulated whole blood. The healthy subjects in the control group had no infectious diseases; liver function, kidney function, and chest imaging examinations were normal; the sputum tuberculosis smear microscopy report was negative, and there was no history of tuberculosis. At the same time, 40 cases of pulmonary tuberculosis patients were treated in the First Affiliated Hospital of Xinjiang Medical University, and they undergo surgical treatment from June 2018 to June 2021; their surrounding lungs and lung stumps were collected.

Patients with typical clinical manifestations and signs of tuberculosis such as low-grade fever, night sweats, and exertion which at least meet one of the following 3 items are active tuberculosis: (1) sputum smear microscopy or sputum culture was MTB positive; (2) sputum examination was negative, and chest imaging examination suggested typical active pulmonary tuberculosis; and (3) sputum test was negative, but pathological diagnosis of pulmonary lesions or pleural fluid and bronchoalveolar lavage fluid was reported as tuberculous lesions. Patients were excluded from other immune diseases, neoplastic diseases, pulmonary infection, and HIV infection. This study was approved by the Ethics Committee of Xinjiang Medical University, and all study subjects were informed and agreed to participate in this study.

### 2.2. Hematoxylin and Eosin (H&E) Staining

Paraffin-embedded tissue sections were dewaxed, stained with hematoxylin for 1 min, stained with eosin for 3 min and 1% hydrochloric acid ethanol for 1 s, and returned to blue with PBS. Dehydrated, dried, and sealed with neutral gum, and the pathological manifestations of the lesions were observed under a microscope.

### 2.3. Immunohistochemistry (IHC) Staining

After dewaxing, the tissue sections were repaired with sodium citrate solution for 15 min and endogenous peroxidase blocker to avoid light conditions at 37°C for 10 minutes. Goat serum was sealed for 20 min and incubated with primary antibody in refrigerator at 4°C for 14 h (Table 1). Combined with secondary antibody at 37°C for 90 min, the staining was performed with diaminobenzidine in peroxide substrate solution for 2.5 min ± 10 s. Image-Pro Plus version 8.0.1 is used to process the collected images of immunohistochemistry results; after quantitative processing (IOD (sum)/Area(sum)), the positive rate is calculated.

### 2.4. Original Data Acquisition

ArrayExpress database is a common repository of microarray gene expression data under EBI; “tuberculosis” was searched in the public database (https://www.ebi.ac.uk/arrayexpress/) we downloaded; Mahdad Noursadeghi uploaded the blood transcriptome data E-MTAB-4257 in October 2016 and collected 49 cases of active tuberculosis and 26 healthy controls. Because the data set and annotation files were downloaded from public databases, patient consent and IRC approval are not required.

The STRING database (https://string-db.org/) is a database for searching known protein interaction relationships. It is used to study protein interaction relationships; it can provide data and predictive tools for protein interactions. We analyzed the important genes adjacent to the relationship between IL-27, CXCL9, CXCL10, and CXCL11 and the protein interaction map predicted by the relationship between them.

### 2.5. Data Process

The RStudio software (v4.0.3) was used to standardize the blood transcriptome data E-MTAB-4257. Gene Set Enrichment Analysis (GSEA) is a tool for analyzing genome-wide expression profiling chip data and uploading all gene sets to the GSEA software (v4.1.0) for enrichment analysis. Normalized enrichment score (NES) > 2, false discovery rate (FDR) < 0.05, and normal *P* < 0.01 were considered statistically significant [18]. Weighted gene coexpression network analysis (WGCNA) is a systematic biological method used to describe correlation modes between genes and find highly correlated modules. The weighing coefficient *β* is determined according to the principle of scaleless network; that is, the correlation coefficient between the logarithm of the number of connected nodes log (*k*) and the logarithm of the probability of occurrence of the node log(*p*(*k*)) is at least 0.8 [19]. Used the limma package to screen the differentially expressed genes between 49 active pulmonary tuberculosis patients and 26 healthy controls, adjusted *P* value < 0.05, and ∣log2 (fold − change) | >1 [20], eligible genes were included in the differentially expressed genes set (DEGs), and a volcano map was drawn. Metascape (http://metascape.org/gp/index.html) is widely used, because they update the database every month. So DEGs were imported into the Metascape online website and RStudio software for Gene Ontology (GO) and Kyoto Encyclopedia of Genes and Genomes (KEGG) pathway enrichment analysis [21] and got the protein interaction map drawn by Metascape online website.

The important gene sets (IGs) involved in IL-27 and CXCL9, CXCL10, and CXCL11 were obtained, through the online STRING database [22]. And GO/KEGG analysis and protein interaction mapping were also performed on the Metascape online website and the RStudio software.

DEGs and IGs were intersected to obtain important target gene sets (TGs) and describe the correlation between the target genes and used the RStudio software to draw a chord diagram and heat map (*P* < 0.05). miRWalk, TarBase v.8, miRDB, miRSystem, and TargetScanHuman 7.1 contain the miRNA target gene information of multiple species such as humans, mice, rabbits, dogs, and fruit flies. Used the TGs predict miRNA, and draw the Venn diagram from five databases prediction information, and get the ultimate key miRNA. The Cytoscape software (v3.8.0) was used to map the interaction between miRNA and target gene picture.

### 2.6. Quantitative Reverse Transcription Polymerase Chain Reaction (qRT-PCR)

5 ml peripheral blood was collected and treated with erythrocyte lysate, to obtain the remaining white blood cells (WBC). Total RNA of peripheral blood leukocytes was extracted with TRIzol solution (Invitrogen, US), and RNA concentration was measured.

IL-27, CXCL9, CXCL10, and CXCL11 were reverse transcribed to synthesize cDNA according to the qRT-PCR kit (Takara, Japan, Code No. RR037A), and then according to the instructions, adding specific primers, and preparing a 25 *μ*l PCR reaction system (Code No. RR820A). GAPDH was an internal reference. The qRT-PCR operation of miRNA is as follows. The RNA molecule is polyadenylated and reverse transcribed using poly(A) polymerase and SMART® MMLV reverse transcriptase in the kit (Cat. No. 639676). TB Green Advantage ® qPCR Premix and mRQ 3′ Primer were used together with miRNA primers for real-time qRT-PCR to quantify specific miRNA sequences in cDNA (Cat. No. 638315). Primer sequences are shown in Table 2.

The delta-delta cycle threshold method (△△Ct) is an approximate method. It compares the sample with the second standardized RNA (such as GAPDH and U6), to measure the relative level of RNA or miRNA in the samples. In this study, the comparative cycle threshold method (2^-△△Ct^) was used for analysis.

### 2.7. Statistical Analysis

The GraphPad Prism (v8.0.1) and RStudio (v4.0.3) software were used for calculation and visualization. For data that does not follow a normal distribution, the Wilcoxon test was used to analyze the differences of variables. Pearson correlation coefficient was used to evaluate the correlation between numerical variables. *P* value < 0.05 was considered statistically significant.

## 3. Results

### 3.1. Tissue Staining of Pulmonary Tuberculosis (TB) Lesions

**Figure 1:**
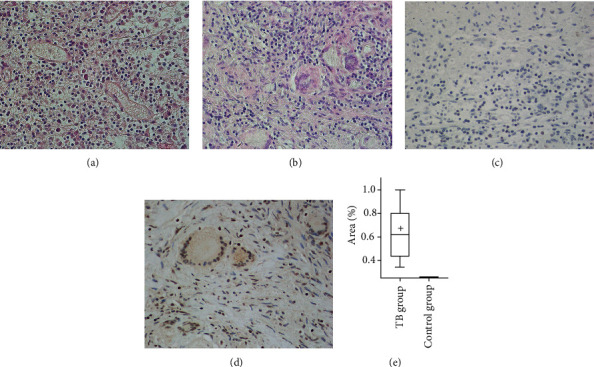
Pulmonary tuberculosis pathological staining. (a, b) Distal side control group and TB group hematoxylin and eosin staining (×200). (c–e) Distal side control and TB group immunohistochemistry staining (×200) and statistical analysis.

After HE staining, the TB lesions showed typical tuberculous granulomas, caseous necrosis accompanied with Langerhans' multinucleated macrophage aggregation. Under the microscope, the cytoplasm was pink, and the blue-purple nuclei are gathered on the edge of giant macrophages like a horseshoe (Figure 1(a)). Inflammatory infiltration was occasionally seen in the distal part; no obvious granulomatous structure was seen (Figure 1(b)).

Ag85B is a virulence protein expressed by MTB; it is an essential structure involved in the construction of cell walls. Under IHC staining of TB lesions, it showed dark brown under the microscope, especially in Langerhans' cell cytoplasm and nucleus were obviously positive expression (Figure 1(c)). However, there was no positive expression area in the distal tissue (Figure 1(d)). The expression difference between the TB lesion site and the distal site was statistically significant (Figure 1(e), *P* < 0.001).

**Figure 2:**
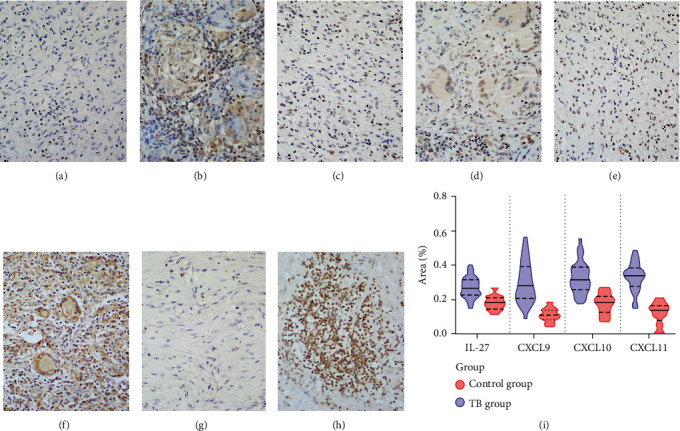
Pulmonary tuberculosis immunohistochemical results. (a, b) Distal side control and TB group interleukin-27 immunohistochemistry staining (×200). (c, d) Distal side control group and TB group CXCL9 immunohistochemistry staining (×200). (e, f) Distal side control group and TB group CXCL10 immunohistochemistry staining (×200). (g, h) Distal side control group and TB group CXCL11 immunohistochemistry staining (×200). (i) Interleukin-27 and CXCL9/10/11 immunohistochemistry staining statistical analysis.

The IHC staining results of IL-27, CXCL9, CXCL10, and CXCL11 were shown in Figure 2. IL-27 was mainly expressed in the cytoplasm of cells; the distal side control group could be seen occasionally expressed in the nucleus. The cytoplasm and nucleus of the granuloma in the TB group showed clear positive expression (Figures 2(a) and 2(b)). CXCL9, CXCL10, and CXCL11 were all clearly positive in the TB lesion, mainly in the nucleus and cytoplasm. However, in the distal side tissues, star-shaped tan areas were seen, mainly in the nucleus (Figures 2(c)–2(h)). Among them, CXCL10 was the most abundant, and CXCL11 is mainly concentrated in the nucleus. In the TB lesion tissues and distal side tissues, the expression difference was statistically significant (Figure 2(i)).

### 3.2. DEG Data Analysis

**Figure 3:**
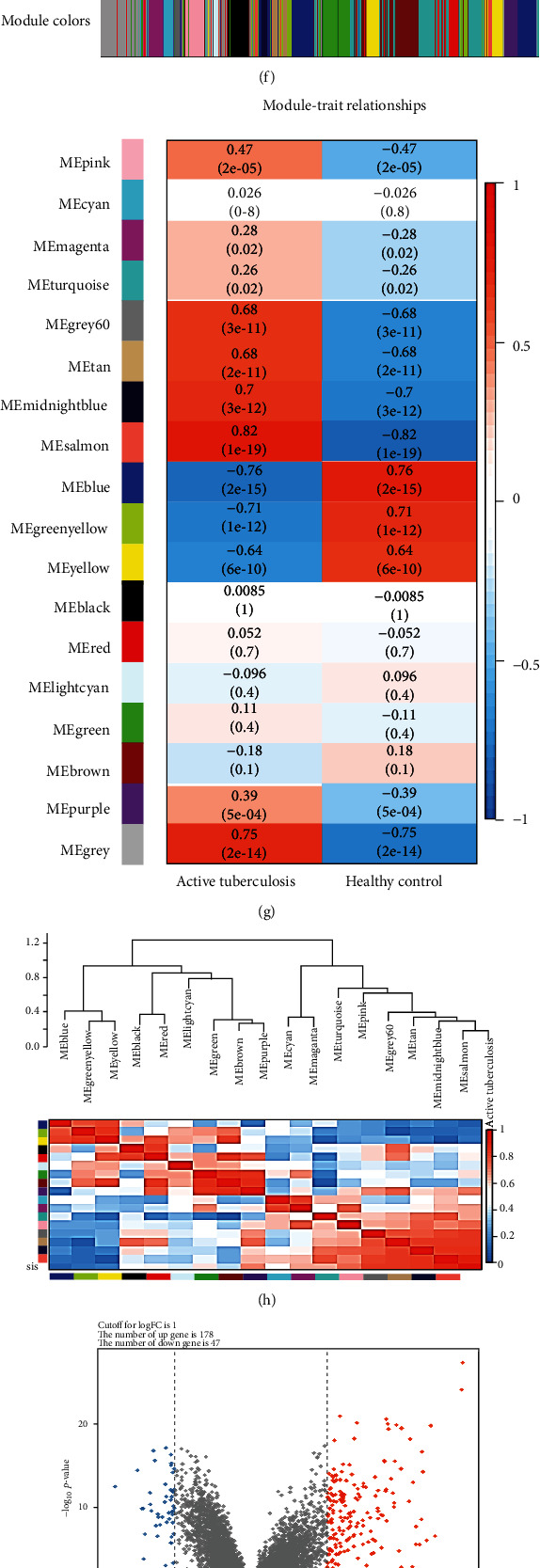
Data set E-MTAB-4257 difference analysis and enrichment analysis. (a–d) Gene Set Enrichment Analysis. (e–h) Weighted gene coexpression network analysis. (i) Differential gene volcano map. (j–l) Gene Ontology and Kyoto Encyclopedia of Genes and Genomes pathway enrichment analysis.

In the data E-MTAB-4257, all gene expressions of 49 active PTB patients and 26 healthy control (HC) patients were analyzed by GSEA; IFN-*γ* signaling pathway, inflammatory response pathway, and IL-6-JAK-STAT signaling pathway were significantly enriched (Figures 3(a)–3(d)). We conducted another WGCNA analysis (Figures 3(e)–3(h)). However, we have not been able to explore clinically significant genes. Therefore, we analyzed the difference between the PTB group and the HC group, and obtained 225 genes, 178 upregulated genes, and 47 downregulated genes (Figure 3(i)). Subsequently, DEGs were mainly enriched in the signaling pathways involved in T cell receptors (Figure 3(j)), antibacterial and antiviral responses (Figure 3(l)), and cytokine pathways (Figure 3(k)), and the Metascape online website was used to draw PPI maps (Figure 3(l)).

### 3.3. IL-27-, CXCL9-, CXCL10-, and CXCL11-Related Gene Exploration

**Figure 4:**
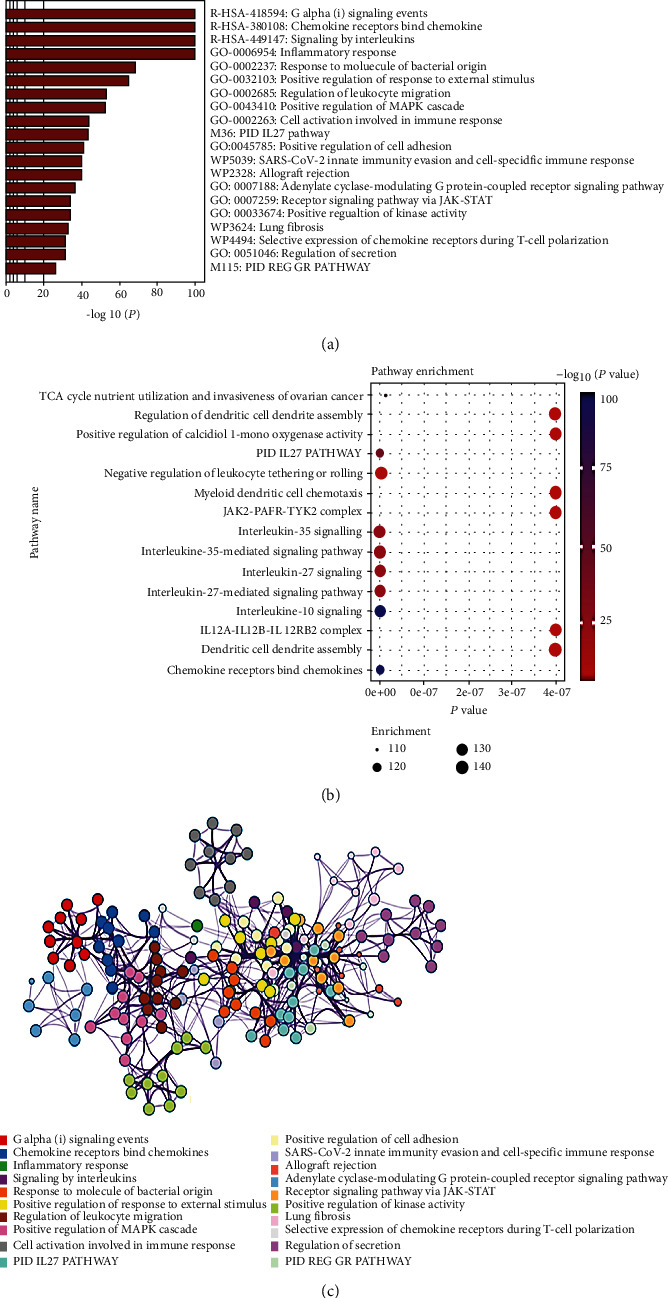
Interleukin-27 and CXCL9/10/11-related gene enrichment analysis. (a–c) Gene Ontology and Kyoto Encyclopedia of Genes and Genomes pathway enrichment analysis.

Since DEG contains 225 genes, we used the STRING database to expand 204 important genes, taking IL-27, CXCL9, CXCL10, and CXCL11 as the key genes. We also performed enrichment analysis and PPI network analysis to verify that these related genes are still important genes involved in chemokines and interleukins (Figure 4).

### 3.4. Predicted miRNA

**Figure 5:**
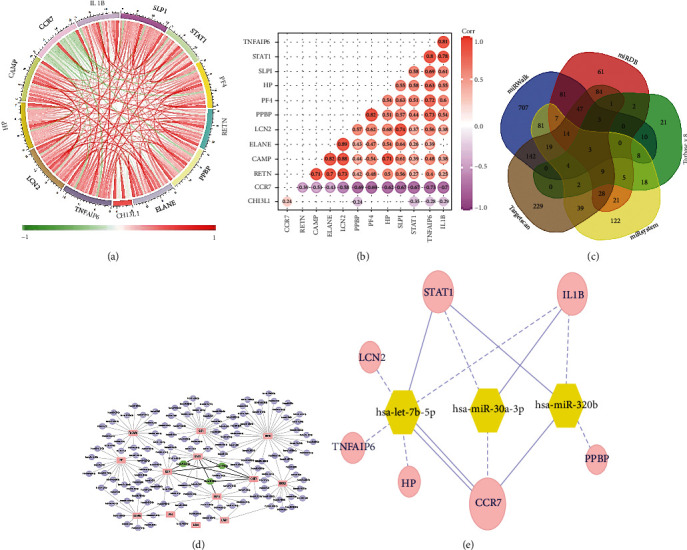
Explore key genes and predict miRNA. (a, b) Correlation analysis of 13 key genes. (c) Venn diagram filter miRNA. (d, e) miRNA and gene interaction.

In the intersection of 225 DEGs and 204 IGs, 13 important target genes (TGs) were obtained (Figures 5(a) and 5(b)). Among them, CCR7 and CHI3L1 were downregulated genes; it was obviously negatively correlated with other genes. And the rest are upregulated genes. Correlation analysis showed that LCN2 was highly positively correlated with ELANE and CAMP, and the correlation coefficients were 0.89 and 0.88 (Figure 5(b)).

1140 miRNAs predicted based on 13 important target genes (TGs). Take the intersection to get 3 key miRNAs (Figure 5(c)), has-let-7b-5p, has-miR-30a-3p, and has-miR-320b. The PPI network diagram was drawn to show their association with 13 target genes (Figure 5(d)) and further describe the genes that are highly related to them (Figure 5(e)). Among them, CCR7 is a downregulated gene; it was closely associated with 3 key miRNAs.

### 3.5. qRT-PCR Results

**Figure 6:**
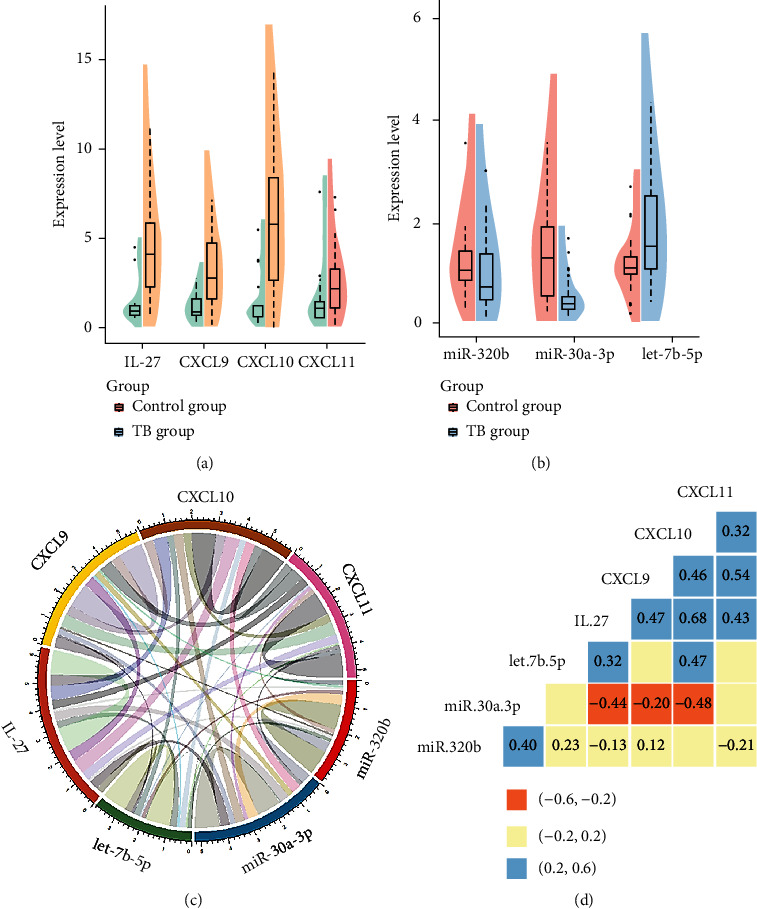
qRT-PCR results. (a) Expression of interleukin-27 and CXCL9/10/11. (b) Expression of miR-320b, mir-30a-3p, and let-7b-5p. (c, d) Correlation analysis.

The qRT-PCR results of IL-27, CXCL9, CXCL10, and CXCL11 were showed; these cytokines were significantly increased in the peripheral blood of TB patients (*P* < 0.05). Compared with the HC group, CXCL10 and IL-27 were most obvious; the difference in CXCL11 expression was not obvious (Figure 6(a)).

3 key miRNAs in the peripheral blood with the TB group are shown in Figure 6(b). let-7b-5p in the TB group was significantly increased (*P* < 0.05), and miR-30a-3p expression was significantly decreased in the TB group (*P* < 0.05). There was no significant difference in the miR-320b expression between the TB group and the control group (*P* > 0.05).

### 3.6. Correlation Analysis

We analyzed the correlation between the PCR results of 4 cytokines and 3 key miRNAs (Figures 6(c) and 6(d)), among which miR-30a-3p was negatively correlated with IL-27 and CXCL10 (*r* = 0.4, *P* < 0.05); in addition, IL-7 has a significant positive correlation with CXCL10 (*r* = 0.6, *P* < 0.05).

## 4. Discussion

In this study, we investigated the expression of IL-27, CXCL9, CXCL10, and CXCL11 in the TB patient lesion tissue and peripheral blood and explore 204 genes related with these indexes. Subsequently, through bioinformatics analysis, the blood transcriptome data of the PTB group and HC group were investigated, and 225 differential genes were explored. After taking the intersection, we found that there were 13 key genes, including 2 downregulated genes, and others were upregulated genes. During the TB pathogenesis, these genes were thought to be involved in IL-27 and CXCL10-mediated antimicrobial processes. Subsequently, we further found out 3 key miRNA, which are has-Let-7b-5p, has-miR-30a-3p and has-miR-320b. PCR experiments were used to verify their expression levels in peripheral blood of TB patients. The expression of let-7b-5p in the TB group was significantly increased, and the expression of miR-30a-3p in the TB group was significantly decreased. Therefore, we believed that Il-27 and CXCL10 play an important role in the prevention MTB, and let-7b-5p and miR-30a-3p are likely to participate and regulate this process.

Interleukin-27 (IL-27) belongs to the IL-12 cytokine family. It consists of two independent subunits p28 and EBI3, mainly secreted by antigen-presenting cells (APCs), such as macrophages and dendritic cells [9, 10]. For TB, IL-27 has a good performance in terms of diagnostic accuracy and improving patient prognosis [23, 24]. In our study, IL-27 was significantly increased in both IHC and qRT-PCR experiment results (Figure 2); therefore, it is reasonable to believe that IL-27 is an important substance involved in TB. CXCL9, CXCL10, and CXCL11 belong to the CXC chemokine subfamily; they are located on human chromosome 4q21 and produced by immunocyte. After they bind to the CXCR3 receptor, they can promote monocytes and lymphocytes to the lesion, thereby exerting cellular immune effect. Among them, the relationship between CXCL10 and TB has been widely concerned [25, 26]. Many scholars believe that CXCL10 is an important biomarker of TB [27]; this is consistent with our research results. However, what is more noteworthy in this study is that in TB patients' peripheral blood, IL-27 and CXCL10 have a certain correlation (*r* = 0.68, *P* < 0.05). Therefore, we believe that IL-27 and CXCL10 play an important role in the process of antituberculosis.

Bioinformatics research is a good way to explore the pathogenesis of diseases. In this study, in order to further explore the role of IL-27 and CXCL10 in tuberculosis, we continue to use bioinformatics methods to combine the prediction results of miRWalk, TarBase, miRDB, miRSystem, and TargetScanHuman. 3 miRNAs were extracted and verified by qPCR experiments (Figure 6(b)). Our study shows that let-7b-5p has a positive correlation with IL-27 and CXCL10; miR-30a-3p is significantly downregulated and has a negative correlation with IL-27 and CXCL10 (Figure 6(d)).

In fact, miR-let-7b-5p research is very limited currently; however, some scholars have noticed its role in infection and inflammation [28, 29]. Our study also suggests that miR-let-7b-5p can participate in the antibacterial process, through the STAT1 pathway (Figure 5(e)). Now, there are studies which pointed out that IL-27 can inhibit viral infection by activating STAT1/3 and CXCL9/10 [30]. However, remarkable, during TB pathogenesis, miR-let-7b-5p may participate and regulate IL-27 and CXCL10 antibacterial process, through STAT1 pathway. This laid the foundation for us to further explore the relationship between IL-27 and CXCL10. In addition, miR-30a-3p was negatively correlated with IL-27 and CXCL10 (Figure 6(d)); this also attracts our attention. In recent years, miR-30a-3p has made some progress in tumors [31–33], and it also has a certain research basis for asthma and pulmonary fibrosis [34, 35]. However, in this bioinformatics study on TB, we discovered 13 key genes involved in the network relationship between IL-27 and CXCL10. Among them, CCR7 was a very important downregulated gene (Figure 5(b)), and miR-30a-3p in TB patient peripheral blood showed a significant downward trend, revealing the close relationship between CCR7 and miR-30a-3p. Therefore, we have reason to believe that miR-30a-3p can negatively regulate the expression levels of IL-27 and CXCL10 through the signal path involved in CCR7. And then, they play an important role in the anti-TB process.

The global epidemic of TB cannot be ignored, especially in the current environment of COVID-19 pneumonia; this is a new challenge for us. Therefore, finding out new biomarkers is a great significance to us. This study detected the expression of IL-27 and CXCL9/10/11 in the lesion tissue and peripheral blood of TB patients and preliminarily revealed the relationship between let-7b-5p and miR-30a-3p with IL-27 and CXCL9/10/11; this provides a certain foundation and new ideas for subsequent research.

Of course, there are deficiencies in this research. When we calculated the correlation between the 3 key miRNAs and IL-27, CXCL9/10/11 through statistical methods, the correlation coefficients were not particularly good. The insufficient sample size was considered an important reason; it is mainly affected by COVID-19 pneumonia. We believe that in the following studies, if the sample size is enlarged, more convincing results will be obtained. In our next research, we will further explore the important roles of let-7b-5p and miR-30a-3p in tuberculosis and their relationship with IL-27 and CXCL10, hoping to have a better understanding on resistance M. TB process.

## Figures and Tables

**Table 1:** Immunohistochemical antibody.

Antibody	Brand	Concentration
Ag85B	Bioss, China	1 : 200
IL-27	Affinity, China	1 : 200
CXCL9	Bioss, China	1 : 150
CXCL10	Bioss, China	1 : 200
CXCL11	Affinity, China	1 : 150

**Table 2:** Primer sequences.

Gene name	Sequence	
IL-27	Forward	CGGAGGGAGTTCACAGTCAG
	Reverse	CAGGTGAGATTCCGCAAAGC
CXCL9	Forward	GAAGCAGCCAAGTCGGTTAGTG
	Reverse	AATCATCAGCAGTGTGAGCAGTG
CXCL10	Forward	TGGCATTCAAGGAGTACCTC
	Reverse	TTGTAGCAATGATCTCAACACG
CXCL11	Forward	CCATCGGAGTTTACAAAGTGCT
	Reverse	TCTCCACCGTAACCACAGATAGT
GAPDH	Forward	CATCCACTGGTGCTGCCAAGGCTGT
	Reverse	ACAACCTGGTCCTCAGTGTAGCCCA
hsa-miR-320b	5′ →3′	TAAAAGCTGGGTTGAGAGGGC
hsa-miR-30a-3p	5′ →3′	CTTTCAGTCGGATGTTTGCAGC
hsa-let-7b-5p	5′ →3′	ATGGGGTGAGGTAGTAGGTTG

## Data Availability

The data used to support the findings of this study are included in the article.
